# Optimizing the frequency of physician encounters in follow - up care for patients with type 2 diabetes mellitus: a systematic review

**DOI:** 10.1186/s12875-024-02277-9

**Published:** 2024-01-26

**Authors:** Wanchun Xu, Ivy Lynn Mak, Ran Zhang, Esther Yee Tak Yu, Amy Pui Pui Ng, David Tak Wai Lui, David Vai Kiong Chao, Samuel Yeung Shan Wong, Cindy Lo Kuen Lam, Eric Yuk Fai Wan

**Affiliations:** 1 Department of Family Medicine and Primary Care, Li Ka Shing Faculty of Medicine, The University of Hong Kong, Hong Kong SAR, China; 2 Department of Medicine, Li Ka Shing Faculty of Medicine, The University of Hong Kong, Hong Kong SAR, China; 3 Department of Family Medicine and Primary Health Care, United Christian Hospital & Tseung Kwan O Hospital, Kowloon East Cluster, Hospital Authority, Hong Kong SAR, China; 4 School of Public Health and Primary Care, The Chinese University of Hong Kong, Hong Kong SAR, China; 5 Centre for Safe Medication Practice and Research, Department of Pharmacology and Pharmacy, Li Ka Shing Faculty of Medicine, The University of Hong Kong, Hong Kong SAR, China

**Keywords:** Type 2 diabetes mellitus, Follow - up care, Physician encounters, Risk stratification

## Abstract

**Background:**

Decisions on the frequency of physician encounters for patients with type 2 diabetes mellitus (T2DM) have significant impacts on both patients’ health outcomes and burden on health systems, whereas definitive intervals for physician encounters are still lacking in most clinical guidelines. This study systematically reviewed the existing evidence evaluating different frequencies of physician encounters among T2DM patients.

**Methods:**

Systematic search of studies evaluating different visit frequencies for follow - up care in T2DM patients was performed in MEDLINE Ovid, Embase Ovid, and Cochrane library from database inception to 25 March 2022. Studies on the follow - up encounters driven by non - physicians and those on the episodic visits in the acute care settings were excluded in the screening. Citation searching was conducted via Google Scholar on the identified papers after screening. The risk of bias was assessed using Cochrane RoB2 tool for randomized controlled trials and Newcastle - Ottawa Scale for cohort studies. Findings were summarized narratively.

**Results:**

Among 6363 records from the database search and 231 references from the citation search, 12 articles were eligible for in - depth review. The results showed that for patients who had not achieved cardiometabolic control, intensifying encounter frequency could enhance medication adherence, shorten the time to achieve the treatment target, and improve the patients’ quality of life. However, for the patients who had already achieved the treatment targets, less frequent encounters were equivalent to intensive encounters in maintaining their cardiometabolic control, and could save considerable healthcare costs without substantially lowering the quality of care and patients’ satisfaction.

**Conclusion:**

Existing evidence suggested that the optimal frequency of physician encounters for patients with T2DM should be individualized, which can be stratified by patients’ risk levels based on the cardiometabolic control to guide the differential scheduling of physician encounters in the follow - up. More research is needed to determine how to optimize the frequency of physician encounters for this large and heterogeneous population.

**Supplementary Information:**

The online version contains supplementary material available at 10.1186/s12875-024-02277-9.

## Introduction

Type 2 diabetes mellitus (T2DM) is a highly prevalent chronic disease worldwide, which usually leads to multiple complications over time, such as cardiovascular diseases [1]. Approximately 462 million individuals were affected by T2DM in 2017, which corresponded to 6.28 % of the world’s population [2]. The global prevalence and direct health expenditure related to diabetes is projected to be 10.2 % and 825 billion USD respectively in 2030 [3, 4]. Physician - patient encounters play important roles in the management of T2DM, including tracking the effect of medication, giving expert guidance on self - care, monitoring the patient’s overall health status, and starting early prevention and treatment for potential complications [5]. Regular physician - patient encounters are recommended in the clinical guidelines from major advisory bodies worldwide to monitor the disease progression and prevent diabetes complications in T2DM patients, including the American Diabetes Association (Standards of Medical Care in Diabetes, 2022) and the National Institute of Health and Care Excellence (Type 2 diabetes in adults: management, 2015) [6, 7]. Decisions on the frequency of physician - patient encounters for this large population group have significant impacts on both patients’ health outcomes and the clinical burden on the health systems. Worldwide concerns regarding the increasing clinical burden of T2DM have prompted healthcare providers and policymakers to rethink the optimal physician - patient encounter frequency for this large patient population.

However, few clinical guidelines have yet to provide definitive recommendations on the appropriate interval for physician - patient encounters. Only fragmented recommendations on the monitoring intervals for some key clinical parameters were given in the existing guidelines, such as the monitoring for HbA1c [7]. According to the guideline established by the American Diabetes Association [6], follow - up visits for the comprehensive medical evaluation should occur at least every 3 - 6 months individualized to patients, and then at least annually. However, it remains unclear how the follow - up interval can be individualized, and most clinical guidelines worldwide do not provide specific recommendations on the defined intervals for physician encounters. This absence of specific recommendations leaves service providers uncertain about the appropriate encounter interval for patients with T2DM. The conventional practice in scheduling physician encounters is usually based upon the physicians’ discretion and influenced by the patient’s preference as well as the resource restriction of the local health systems [8–10].

Given the considerable number and heterogeneity among patients with T2DM, the optimization in the demand for physician consultation would significantly improve the cost - effectiveness of diabetes care from various stakeholder perspectives. The concept of optimizing diabetes care through risk stratification among patients with T2DM has attracted an increasing amount of attention and evidence support worldwide [11, 12]. Theoretically, the revisit interval could be individualized based on the patient’s health status and risk for complications. Previous studies on different types of diabetes patients also revealed the necessity of differentiating encounter frequencies in this population, where equivalence in glycemic control was reported between different follow - up frequencies among the patients under optimal control [13], and increased encounter frequency showed benefits in achieving treatment target for patients with suboptimal control in some other studies [14, 15]. However, the research on the optimization of the physician encounter frequency for type 2 diabetes patients is still in its infancy and the existing research evidence on this topic has not been reviewed. Previous studies on this topic also differed in terms of patient population, sample size, outcome measures and other methodological factors, yielding inconclusive results. A systematic review of the available evidence on the optimal frequency of physician encounters for T2DM patients is needed to gather the existing knowledge to assess the quality of the existing evidence and provide a comprehensive conclusion, in addition to identifying the knowledge gaps that require further studies. Therefore, this study aimed to conduct a systematic review of existing evidence comparing the quality and effectiveness of care across different frequencies of physician - patient encounters among patients with T2DM.

## Method

### Criteria for considering studies for this review

#### Types of participants

Studies on adult patients with T2DM, including those with comorbidities, are included in this review. The studies involving patients with type 1 diabetes (T1DM) and gestational diabetes mellitus (GDM) were excluded from the review.

#### Types of interventions

Studies comparing different frequencies of physician encounters were included, with no restriction on the type of setting (i.e., primary, secondary and tertiary medical institutes). To avoid missing the potential studies, the database search included all studies evaluating different visit frequencies for the patients with T2DM without any restriction to the service providers. Only the records with a study design that involved physician - patient encounters were considered at the screening stage to ensure comparability. Studies that evaluated the frequency of follow - up care provided by nurses, pharmacists, or other non - physician health practitioners were excluded at the stage of screening. Studies on the episodic visits to the health sectors in the acute care settings (e.g., urgent care or emergency department) were also excluded. Only RCTs or cohort studies that examined the association between encounter frequencies and the subsequent outcomes were eligible for this review. Cross - sectional studies were excluded due to their inability to establish causal inferences.

#### Types of outcome measures

The outcomes of interest included all potential indicators for evaluating the quality and effectiveness of diabetes care, including the main clinical parameters reflecting cardiometabolic control (blood glucose, blood pressure and blood lipids profile), the incidence of diabetes complications or mortality rate as long - term health outcomes, quality of care, cost - effectiveness, patients’ satisfaction, quality of life, and other outcomes related to diabetes care.

### Search strategy

A systematic search was conducted in the database of MEDLINE Ovid (1946 to 25 March 2022), Embase Ovid (1974 to 25 March 2022) and Cochrane Library (searched up to 25 March 2022). The search strategy was initially modeled based on the one designed for MEDLINE Ovid, and subsequently adapted for the other databases. The search consisted of terms related to T2DM and follow - up frequency, including the subject headings, subheadings, and text words used to describe the concept clusters (e.g., revisit, encounter, monitor, etc). Proximity operators were used to achieve more specificity and thus reduce the irrelevant references retrieved [16]. The full search strategy with the used keywords is provided in the Supplementary materials (Supplementary method). In addition, citation searching was conducted to further identify potential articles that were not found by database searches. For the key papers identified from MEDLINE Ovid, Embase Ovid and Cochrane Library, we performed searches in Google Scholar to collect papers that have cited these key papers. This review was registered with PROSPERO (CRD42022360845).

### Data collection and analysis

#### Selection of studies, data extraction, and risk of bias appraisal

Two reviewers independently screened the extracted titles and abstracts against the eligibility criteria to identify the potential studies for full - text review. The in - depth review was limited to the peer - reviewed full - text articles in English. Only primary research was considered for the full - text review. Letters, conference abstracts, and study protocols were excluded from the list of studies reviewed. Only randomized control trials (RCTs) or cohort studies were considered for the in - depth review. Cross - sectional studies were excluded from the analysis. Conflicts over study inclusion were resolved by consultation with a third reviewer. A flow diagram of the number of reports identified and excluded at each stage was prepared in accordance with the PRISMA flow diagram [17]. All studies meeting the criteria for in - depth review were subject to data extraction and risk of bias appraisal. We extracted the details of study settings, study design, participants, interventions and control, follow - up duration, and outcomes of interest, as mentioned above. Risk of bias was assessed using the Cochrane RoB2 tool for RCT studies [18] and the Newcastle - Ottawa Scale for cohort studies [19].

#### Data analysis and presentation

We conducted a descriptive summary of the included studies regarding the participants, interventions, comparators, and the results of the study outcomes. The feasibility of the meta - analysis was considered. Given the heterogeneity among the studies in terms of the participants (patients with different statuses of disease control), intervention (a wide range of visit frequencies), and efficacy endpoints (different outcomes or treatment targets), a meta - analysis was not feasible, and instead a descriptive approach was taken to comprehensively synthesize the findings from the literature. Histograms were used to visualize the characteristics of the included studies and the main findings.

Although the studies involving T1DM and GDM were excluded from this review, certain studies that did not specify the type of diabetes were included to prevent the loss of valuable information. To test if the decision on the eligibility of these studies would affect the results, a sensitivity analysis was conducted by further excluding these studies.

## Findings

### Extent of the studies identified

**Fig. 1:**
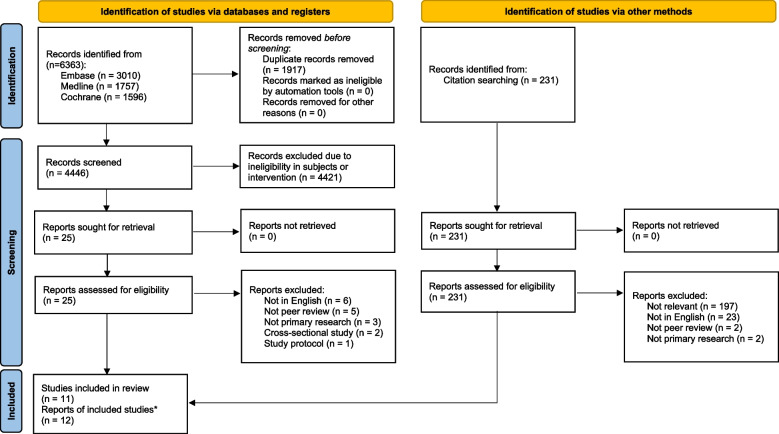
Study flow diagram for each stage of review Notes: * two reports were from the same RCT study

Figure 1 illustrates the study flow. A total of 6363 records were identified from the electronic databases. After removing 1917 duplicate records, 4421 records were excluded due to the ineligibility of the study participants or intervention. Thus, 25 reports were included for full - text review, among which 17 studies were excluded due to the reasons listed in Figure 1. Eight eligible articles were identified from the database search. A total of 231 citations were identified by manual searching in Google Scholar in the citation searching. Four additional articles were considered eligible for this review. Finally, 12 articles from 11 studies were included for the in - depth review (two articles reported the results from the same RCT study).

### Risk of bias assessments

The results of the risk of bias assessment are displayed in Supplementary Figure 1. For the RCT studies, moderate concerns for bias in the randomization process were found in the two papers from the EFFIMODI (Efficient Monitoring of Diabetes) study (Wermeling, 2014; Wermeling, 2013) [13, 20], where only the patients without a preference for monitoring frequency were randomized. Of 9 cohort studies, 8 were of high quality and presented a low risk of bias. The longitudinal study performed by Moradi *et al* was categorized to be with a high risk of bias [21], where only binary logistic regression was conducted without adjustments for potential confounders.

### Study characteristics

**Fig. 2:**
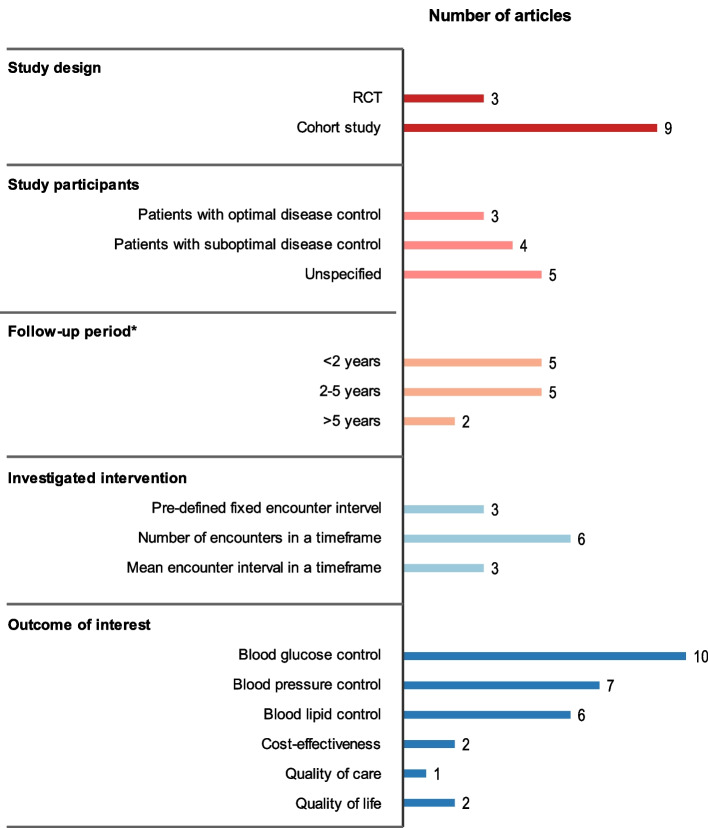
Characteristics of the included studies in the systematic review Note: * The longest follow-up period of the subjects in the reviewed study

The characteristics of the reviewed studies are shown in Table 1 and Figure 2. Large variations in the study design were found among these studies. Three articles were from 2 RCTs, and the remaining 9 articles were from cohort studies. One RCT and one cohort study were conducted on patients with optimal disease control, one RCT and three cohort studies reported the results from the patients with suboptimal disease control, and the remainder were unspecified. The follow - up period of the study subjects was less than 2 years in half of the studies and only two studies had a follow - up period over 5 years. Only three studies (four articles) evaluated pre - defined fixed encounter intervals, and the remaining studies investigated the effect of the numbers of encounters or the mean encounter interval in a timeframe. Blood glucose control was the major outcome of interest (included in 11 out of 13 articles). Other outcomes of interest included blood pressure control, blood lipid control, patients’ quality of life, quality of care, and cost - effectiveness.

**Table 1:** Summary of the findings in the studies identified from systematic review

**Study**	**Country, setting and study design**	**Participants**	**Intervention vs. Comparator**	**Follow - up duration**	**Results on outcome of interest**						
					**Major clinical parameters for diabetes monitoring**					**Cost - effectiveness / minimization**	**Others**
					**Blood glucose**		**Blood pressure**	**Lipid profile**			
**RCTs**											
Wermeling, 2014 [13]	**Country:** Netherlands **Setting:** primary care **Study design:** Pragmatic randomised controlled patient - preference equivalence trial	2215 T2DM patients (with satisfactory HbA1c, SBP and TC control and not on insulin) across the Netherlands included from April 2009 to August 2010	Monitoring frequency: 6 - monthly vs. 3 - monthly	18 months	**Pct. of patients maintaining HbA1c ≤ 7.5 %**:93.3 % vs. 92.0 % (Diff: 1.3 (- 2.4,5.0), equivalent^a^)		**Pct. of patients maintaining SBP ≤ 145 mmHg**: 80.4 % vs. 82.9 % (Diff: - 2.6 (- 8.0, 2.9), uncertain^a^)	**Pct. of patients maintaining TC ≤ 5.2mmol / L**: 92.8 % vs. 91.7 % (Diff:1.0 (- 2.7,4.8), equivalent^a^)		**Cost during study period (euro) Total cost:** 2530.29 ± 8975.01 vs. 2917.51 ± 11797.13 (Diff: - 387) **Direct cost:** 1413.19 ± 2714.46 vs. 1346.99 ± 1605.25 (Diff: 66) **Indirect cost:** 2530.29 ± 8975.01 vs. 2917.51 ± 11797.13 (Diff: - 453)	**Quality of life: SF - 36 physical component scores**: 46.1 ± 10.4 vs. 45.7 ± 10.6 (diff: 0.45 (− 0.78, 1.68), equivalent) **EQ - 5D:** 0.86 ± 0.19 vs. 0.87 ± 0.15 (diff: − 0.01 (− 0.03 to 0.01), equivalent)
					**Pct. of patients remaining HbA1c ≤ 7.5 %, SBP ≤ 145 and TC ≤ 5.2mmol / L:** 69.8 % vs. 69.5 % (Diff: 0.3 (− 6.2, 6.7), uncertain)						
Wermeling, 2013 [20]	**Country:** Netherlands **Setting:** primary care **Study design:** Pragmatic randomised controlled patient - preference equivalence trial		Monitoring frequency: 6 - monthly vs. 3 - monthly	18 months							**Patient satisfaction:** 88.5 % vs. 93.5 %, *p*< 0.001. Patients who determined their own monitoring frequency were more satisfied.
Hu, 2012 [22]	**Country:** China **Setting:** Outpatient follow - up in the hospital **Study design:** a randomized control trial	155 patients with T2DM (with clinical symptom and not meeting blood glucose control) were recruited from December 2009 to December 2010	Follow - up frequency: Monthly vs. 3 - monthly	1 year	**HbA1c:** baseline: 8.10**±**1.61 vs. 8.65 ± 2.2512th month: 5.04 ± 1.15 vs. 6.25 ± 0.58 Repeated - measures analysis at month 3, 6, 9, 12: *P*< 0.05 for overall difference between groups)						**Quality - of - life scale for diabetes Mellitus (DMQLS):** Monthly follow - up showed significant improvement in the disease, psychology and satisfaction domains, as well as the overall DMQLS score, but not for the physiology and society domains.
**Cohort studies**											
Zhao,2022 [23]	**Country:** China **Setting:** General hospitals and community hospitals (National Metabolic Management Center (MMC) Program) **Study design:** Prospective, multi - center, cohort study	19908 participants with T2DM recruited in the MMC between Jun 2017 and April 2021	Follow - up frequency: ≤ 2 times per year vs. > 2 times per year	6 months - 46 months	**Pct. Change of Hba1c from baseline (%):**- 9.67 ± 20.29 vs. - 12.14 ± 19.78 (*p*< 0.0001)		**Pct. Change from baseline (%):** **SBP:** 0.79 ± 14.49 vs.0.13 ± 14.71 (*p*= 0.0004) **DBP:** 0.44 ± 14.40 vs. - 0.28 ± 14.98 (*p*= 0.0006)	**Pct. Change from baseline (%):** **Triglyceride:** 6.32 ± 79.01 vs. 3.79 ± 80.05 (*p*= 0.025) **TC:** - 1.62 ± 25.60 vs. - 2.39 ± 26.21 (*p*= 0.0048) **HDL - c:** 10.69 ± 73.79 vs. 8.58 ± 38.53 (*p*= 0.0094) **LDL - c:** - 4.01 ± 44.91 vs. - 5.07 ± 38.56 (*p*= 0.037)			**Pct. Change from baseline (%):** **Fasting C - peptide:** 19.65 ± 316.91 vs. 19.12 ± 140.39 (*p*= 0.89)
Ye, 2021 [24]	**Country:** The U.S. **Setting:** Primary care (Christie Clinics) **Study design:** Retrospective cohort study	2124 patients with T2DM from 95 ZIP codes with records for more than 80 % of the observation timeframe, from 02 / 01 / 2013 to 12 / 21 / 2015.	Number of physical encounters in each 6 - month period during the study (Mean: 3.97, SD:6.03)	35 months	**One additional physical encounter in a 6 - month period marginally increases the probability of transition**: from diabetic state (BG > 125mg / dl) to a pre - diabetic state (BG 100–125mg / dl) by 4.3 % ; from pre - diabetic to the non - diabetic state (BG < 100mg / dl) by 3.2 % .						
Ukai, 2019 [25]	**Country:** Japan **Setting:** Outpatient follow - up in Specific Health Checkups program **Study design:** Retrospective cohort study	Among 12,145 T2DM patients with satisfactory HbA1c control from 2011 to 2014, 693 with monthly follow - up and 693 with bimonthly follow - up were matched.	Follow - up interval: Bimonthly vs. monthly	1 year	**Pct. of patients maintaining HbA1c ≤ 7.0 %:** 95.0 % vs. 94.4 % (diff: 0.6 (- 1.8, 2.9), equivalent^a^).		**Pct. of patients maintaining SBP ≤ 140 and DBP ≤ 90 mmHg):** 73.3 % vs. 74.2 % (diff: - 0.9 (- 5.5, 3.9), equivalent^a^)	**Pct. of patients maintaining LDL ≤ 140, HDL ≥ 34, and TG ≤ 300 mg / dl:** 81.2 % vs. 81.8 % (diff: - 0.6 (- 4.7, 3.5), equivalent^a^)		**Annual cost for diabetes care:** 96,292 (SD: 179,272) JPY vs. 161,294(SD: 151,826) JPY (diff: - 6500.17 (- 8253.53, - 4746.80))	
					**Pct. of patients achieving all these targets**: 56.9 % vs.58.6 % (diff:- 1.7 (- 6.9, 3.5), uncertain)						
Dobbins, 2019 [26]	**Country:** The U.S. **Setting:** Primary care **Study design:** Retrospective cohort study	7106 newly - diagnosed T2DM patients between July 1, 2012 and June 30, 2013	Number of encounters during exposure period^b^ (Mean:5.8, SD:3.5)	36 months	For patients not achieving HbA1c control (< 8.0 %) at baseline: **OR of HbA1c control during outcome period**^**b**^**:** 1.06 (1.00, 1.12)						For patients with nonadherent in exposure period: **OR of non - insulin diabetes medication adherence in the outcome period**^**b**^**:** 1.12 (1.10, 1.15)
Moradi, 2017 [21]	**Country**: Iran **Setting**: Diabetes clinics **Study design:** Retrospective cohort study	498 patients with T2DM followed in the diabetes clinics between 2006 and 2011	Mean number of visits per year during follow - up period (mean: 2.6, range:0.5 - 7.5)	3 - 5 years	**OR of achieving target HbA1c** with every additional visit per year **(< 7 %):** 0.99 (0.81, 1.23)	**OR of achieving target BP (< 140 mm / Hg)** with every additional visit per year: 0.86(0.68, 1.10)			**OR of achieving target** **LDL (< 70 mg / dl)** with every additional visit per year**:** 0.90 (0.73,1.11)		
Asao, 2014 [14]	**Country:** The U.S. **Setting:** Primary care **Study design:** Prospective, multi - center, cohort study	6,040 eligible adult participants with T2DM (not bed - bound) whose primary care providers were the main provider of the diabetes care	An annual revisit count of outpatient visits categorized into 4 quartiles (times / yr): Q1: < 3.7 Q2: 3.7 - 4.0 Q3: 4 - 6.0 Q4: > 6.0	18 months	Compared to Q1, the predicted marginal prevalence ratio of achieving target HbA1c (< 8.5 %/< 9.5): Q2: 1.03 (0.98, 1.09) / 1.01 (0.97, 1.06) Q3: 1.08 (1.02, 1.15) / 1.07 (1.02, 1.11) Q4: 1.13 (1.04, 1.22) / 1.08 (1.02, 1.14)	Compared to Q1, the predicted marginal prevalence ratio of achieving target BP (< 130 / 85 and 140 / 90 mmHg): Q2: 1.02 (0.91, 1.15) / 1.06 (0.98, 1.14) Q3: 1.03 (0.89, 1.21) / 1.05 (0.97, 1.14) Q4: 1.08 (0.94, 1.24) / 1.05 (0.97, 1.14)			Compared to Q1, the predicted marginal prevalence ratio of achieving target LDL - c (< 100 and < 130 mg / dL): Q2: 1.05 (0.92, 1.20) / 1.03 (0.98, 1.09) Q3: 1.15 (0.98, 1.34) / 1.07 (0.99, 1.16) Q4: 1.20 (1.03, 1.39) / 1.08 (1.02, 1.14)		**Quality of care:** The likelihood of participants who had annual assessments of HbA1c and LDL - c, foot examinations, aspirin use, and influenza immunizations were slightly higher for those with higher revisit frequency (quartiles).
Egan, 2012 [15]	**Country:** The U.S. **Setting:** Primary care **Study design:** Retrospective cohort study	22,285 adults with T2DM at 110 outpatient clinics in the Southeast U.S. from 2004–2008.	Number of annual healthcare visits	4 years	**OR of HbA1c control to < 7 %:** 1.13(1.12 - 1.15)						
Morrison, 2011 [27]	**Country:** The U.S. **Setting:** primary care (affiliated with the hospitals) **Study design:** Retrospective cohort study	26,496 patients with diabetes and elevated HbA1c,BP, and / or LDL treated by primary care physicians between 1 / 1 / 2000 and 1 / 1 / 2009.	Mean encounter interval during the uncontrol period (elevated HbA1c,BP, and / or LDL)	2 - 9 years	Doubling the time of encounter interval was associated a 35 % and 17 % increase in **median time to HbA1c target (< 7 %)**, off and on insulin, respectively (*p*< 0.0001).	Doubling the time of encounter interval was associated with an 87 % increase in **median time to blood pressure target (< 130 / 85 mmHg)** (*p*< 0.0001).			Doubling the time of encounter interval was associated with a 27 % increase in **median time to LDL target (< 100mg / dl)** (*p*< 0.0001).		
Turchin, 2010 [28]	**Country:** The U.S. **Setting:** primary care (affiliated with the hospitals) **Study design:** Retrospective cohort study	5,042 Hypertension patients with DM followed by primary care physicians affiliated with 2 academic hospitals for ≥ 2 years between 1 / 1 / 2000 and 31 / 8 / 2005	Average encounter interval during the single hypertensive period^c^ (≥ 130 / 85 mm Hg).	2 - 5.5 years		An increase in 1 month of the average encounter interval was associated with a HR of 0.764 (0.755, 0.774) for **BP normalization**. Average encounter interval ≤ 2 weeks vs. 2 - 4 weeks **Time / months to BP normalization:** 0.7 vs.1.9					

*Abbreviation*: *Pct.* Percentage, *HbA1c* Hemoglobin A1C, *SBP* Systolic blood pressure, *DBP* Diastolic blood pressure, *LDL* Low - density lipoprotein, *HDL* High - density lipoprotein, *TC* Total cholesterol, *TG* Triglycerides, *QOL* Quality of life, *DMQLS* QoL Scale for Diabetes Mellitus, *SF - 36* 36 - Item Short Form Survey (SF - 36) for quality - of - life measures, *EQ - 5D* An instrument which evaluates the generic quality of life developed in Europe, *JPY* Japanese Yen, *diff* Difference, *HR* Hazard ratio

^a^Equivalent: If the entire confidence interval is between the range − 5 and 5, different follow - up frequencies can be considered equivalent. If it is completely outside this range, it is not equivalent and otherwise it is uncertain

^b^Exposure period and outcome period: The exposure period was the 24 months immediately after the index date, and the outcome period was defined as Months 25 - 36 after the index date

^c^Hypertensive period: From the date of the first encounter with BP above the target to the date of the subsequent encounter with BP below the target

### Impact of frequency of physician encounters on various outcomes

**Fig. 3:**
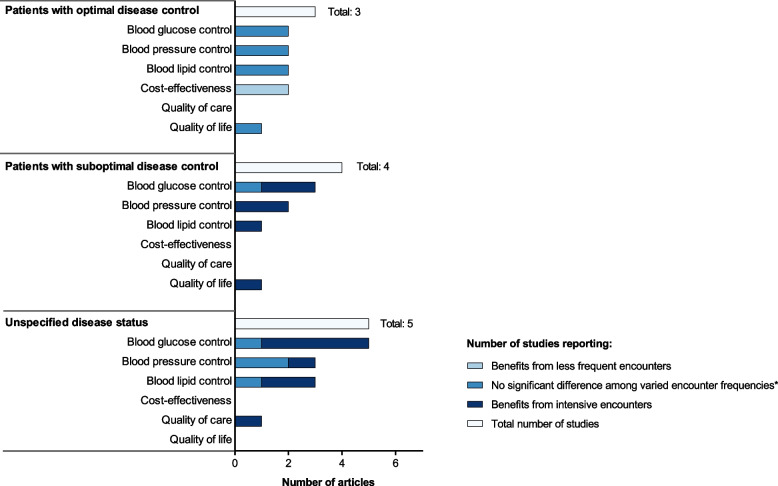
Histogram plot showing the findings on outcomes of interest in the included studies (categorized by patients' status of disease control) Notes: Studies reported no significant difference in the outcome of interest among the patients with varied encounter frequencies

Table 1 and Figure 3 display the results and the details of the study design of the included studies.

#### Main clinical parameters reflecting cardiometabolic control

##### Blood glucose control

Studies have demonstrated the equivalence in maintaining blood glucose control between different encounter frequencies among T2DM patients with optimal control in blood glucose. In RCT by Wermeling *et al* (hereinafter referred to as Wermeling, 2014), 93.3 % and 92.2 % of the patients had Hemoglobin A1C (HbA1c) staying ≤ 7.5 % in the 6 - monthly and 3 - monthly monitoring groups by the end of 18 - month follow - up, respectively (difference: 1.3 %, 95 % CI: (- 2.4 %, 5.0 %)) [13]. Ukai et al. reported similar results in a cohort study, in which 94.4 % of patients in the monthly follow - up group were able to maintain HbA1c ≤ 7.0 % after 1 - year follow - up, versus 95.5 % in the bimonthly (once every 2 months, similarly hereinafter) group (difference: 0.6 %, 95 % CI (- 1.8 %, 2.9 %)) [25]. For the patients with suboptimal disease control at baseline, two studies reported the benefits of the intensive encounter frequencies. In a prospective study of 6,040 T2DM patients in the US (Asao, 2014), the proportion of participants who achieved the HbA1c target (< 9.5 %) after 18 months was slightly higher for those with a higher revisit frequency (over 4 times per year) [14]. Similarly, a retrospective cohort study in the U.S. (Egan, 2012) reported that the increment of one visit per year was associated with a significantly greater likelihood of achieving the HbA1c target of < 7 % [15]. In other studies where the baseline status of patients was not specified, most studies also reported on the benefits of a more intensive encounter frequency. Morrison et al. in a retrospective cohort of 26,496 T2DM patients found that doubling the times of encounter interval was associated with a 35 % and 17 % increase in median time to HbA1c target (< 7 %) among the non - insulin - treated and insulin - treated patients, respectively [27]. A retrospective study on newly diagnosed T2DM patients found that one additional physical encounter within a 6 - month period could increase the transition probability from a diabetic state to a pre - diabetic state by 4.3 % and from pre - diabetic to the non - diabetic state by 3.2 % over the follow - up period [24]. Three studies investigated the outcome of the HbA1c level on a continuous scale, where intensive encounter frequency was found to improve the blood glucose control for patients with suboptimal control or unspecified status (Hu, 2012; Zhao, 2022) [23, 29].

##### Blood pressure control

Equivalence in maintaining blood pressure control was also found in the studies conducted on patients with optimal disease control. In the RCT by Wermeling *et al*, 80.4 % of the patients in the 6 - monthly monitoring group were able to maintain systolic blood pressure (SBP) ≤ 145mmHg, and 82.9 % in the 3 - monthly monitoring group (difference: 1.3 %, 95 % CI: (- 8.0 %, 2.9 %)) [13]. In the cohort study conducted by Ukai *et al*, 74.2 % of patients in the monthly follow - up group were able to maintain SBP ≤ 140 & DBP ≤ 90mmHg, versus 73.3 % in the bimonthly group (difference: - 0.9 %, 95 % CI (- 5.5 %, 3.9 %)). Asao et al. [14] and Moradi et al. [21] also reported the equivalence in blood pressure control among varied encounter frequencies, although different treatment targets for blood pressure were used and the patients’ status of disease control was not specified in these two studies. Zhao et al. reported statistically significant differences in the percentage change of blood pressure control from baseline (*p*< 0.001) between the patients visiting physicians ≤ 2 times per year and those > 2 times per year, but the magnitude of the difference was likely too small to be clinically important [23]. Nonetheless, studies reported that the intensified encounter frequencies benefited the patients with suboptimal blood pressure control at baseline. Turchin et al. found that each increment of 1 month in the encounter interval was associated with a lower chance of achieving blood pressure normalization (target: < 130 / 85mmHg, hazard ratio: 0.764 (0.755, 0.774)) [28], and Morrison et al. reported that doubling the time of encounter interval was associated with 87 % increase in median time to reach blood pressure target (< 130 / 85mmHg) in a retrospective cohort study on 26,496 patients treated by primary care physicians.

##### Blood lipid control

Patients with stable disease control at baseline had comparable maintenance in blood lipid control regardless of follow - up with intensive or with less - frequent encounters (Wermeling, 2014; Ukai, 2019) [13, 25]. Alternatively, the benefits of intensifying the encounter frequency were observed among patients with suboptimal cardiometabolic control at baseline (Morrison, 2011), where doubling the times of encounter interval was related to a 27 % increase in the median time to the low - density lipoprotein (LDL) target of < 100mg / dl (*p*< 0.0001) [27]. For the studies that did not specify the disease control status of the study subjects, two studies reported the benefits of intensive encounters (Zhao, 2022; Asao, 2014) [14, 23], whereas another two studies found no significant difference in blood lipid control among varied encounter frequencies (Moradi, 2017) [21].

##### Overall cardiometabolic control

The overall cardiometabolic control that encompasses the control of blood glucose, blood pressure and blood lipid was also examined in two studies conducted on the patients with stable disease control. In the RCT by Wermeling *et al*, little difference was found in the proportion of patients maintaining overall cardiometabolic control between the 6 - monthly and 3 - monthly monitoring groups: 69.8 % versus 69.5 % (target: HbA1c ≤ 7.5 %, SBP ≤ 145mmHg, and total cholesterol ≤ 5.2mmol / L) [13]. In the cohort study conducted by Ukai et al., the proportion of patients maintaining overall cardiometabolic control was 56.9 % in the bimonthly monitoring group and 58.6 % in the monthly monitoring group (target: HbA1c ≤ 7.0 %, BP ≤ 140 / 90mmHg, LDL ≤ 140mg / dL, high - density lipoprotein ≥ 34mg / dL, and triglycerides ≤ 300mg / dL) [25].

#### Cost - effectiveness

In the RCT by Wermeling *et al*, the cost - minimization analysis found that the total costs incurred for patients that were monitored every 6 months was 387 euros less than those monitored in 3 - month intervals during the study period (18 months), and the indirect cost was reduced by 453 euros [13]. In the cohort study by Ukai *et al*, the bimonthly follow - up was found to be cost - saving in terms of the annual medical costs for diabetes care compared with the monthly follow - up (difference: - 6500.17, 95 % CI: (- 8253.53, - 4746.80), unit: Japanese Yen (JPY)) [25].

#### Other outcomes of interest

With regard to quality of care, Asao et al. found that the proportion of patients receiving annual assessments of HbA1c, LDL, foot examinations, influenza immunization, and aspirin used (advised or documented) was slightly higher among the patients with higher revisit frequency in a prospective cohort study. However, the magnitude of the reported odds ratio was small (close to 1), suggesting that the absolute difference should be minimal [14]. Similarly, among the patients with stable diseaseStandards of Medical Care in Diabetes-2022 control, Wermeling et al found that the patients’ satisfaction with 3 - monthly monitoring was slightly higher than that with 6 - monthly monitoring but the satisfaction with 6 - monthly monitoring was still rather high (93.5 % and 88.5 %, respectively, *p*< 0.001) [20]. The intensive encounter frequency was found to improve patient’s quality of life (QOL, measured by a QOL scale for DM containing items from domains of Diseases, Physiology, Society, Psychology and satisfaction) in a RCT conducted on 155 T2DM patients with suboptimal disease control (Hu, 2012) [22]. Alternatively, there were no differences in QOL (measured by QOL scales of SF - 36 and EQ - 5D) between different monitoring frequencies among the patients with stable disease control (Wermeling, 2014) [13]. Additionally, the frequent encounters with primary care providers were also found to significantly increase non - insulin diabetes medication adherence among newly diagnosed T2DM patients with poor adherence at baseline (OR: 1.12, 95 % CI (1.10, 1.15)) (Dobbins, 2019) [26].

#### Sensitivity analysis

In the sensitivity analysis, two studies (Morrison 2011 and Turchin 2010) were excluded due to lack of specification regarding the type of diabetes in the articles [27, 28]. Both studies focused on patients with suboptimal control, and thus the exclusion of these two studies would not affect the conclusion for the patients with optimal control (Supplementary Figure 2). The synthesis of the remaining two studies (Hu, 2012; Dobbins, 2019) in the same category yielded consistent conclusion with the primary analysis, suggesting benefits for intensifying follow - up frequency for patients with suboptimal control. Hu’s RCT revealed that intensive follow - up frequency is significantly associated with improved HbA1c control and better quality of life (*p*< 0.05) [22]; Dobbins’ cohort study showed a significant association between more frequent encounters with better medication adherence (adjusted OR: 1.12 [1.10, 1.15]), and an insignificant but directional association with improved HbA1c control (OR: 1.06 [1.00, 1.12]) [26].

## Discussion

### Summary of the main results

Despite the great heterogeneity in the settings and methodology, findings from the included studies in our review were informative. First, intensifying the frequency of physician encounters was proved to significantly enhance the patients’ medication adherence, shorten the time to normalization of cardiometabolic risk factors, and improve patients’ QOL during their suboptimal control period. Second, the less - frequent follow - up and the intensive frequencies of physician encounters were found to be equivalent in maintaining cardiometabolic control among the patients who have already achieved the treatment target. Additionally, reducing encounter frequency would not substantially impair the quality of care and patients’ satisfaction, and the cost - saving was estimated to be considerable for patients with optimal cardiometabolic control.

### Discussion on main findings

Intensifying the frequency of physician encounters was found to benefit the T2DM patients during their suboptimal control period in multiple aspects. Physician encounters play an important role in the oversight of patients’ disease progression, timely medication intensification, and informing patients about their problems [24]. Thus, more frequent encounters would improve patients’ treatment strategy and adherence, thereby shortening the time to treatment target and improving the patient’s QOL. Although the benefits of increasing encounter frequency were established among the T2DM patients during the suboptimal control period, the optimal interval for the encounter schedule remained unclear. Increasing the frequency of physician encounters would increase the demand for healthcare resources and burden on healthcare systems, especially for health systems with resource restrictions. Hence, it is crucial to investigate the optimal interval for intensive follow - up to achieve the trade - off between improving health outcomes and minimizing medical costs for patients with suboptimal disease control. Among the four studies on patients with suboptimal disease control in our review, only one study compared the effectiveness of the pre - defined fixed follow - up intervals, where the interval of intensive follow - up was 1 month. Nonetheless, whether the monthly follow - up can be generalized to the population level or is feasible in different contexts remains unknown. Future studies on the optimal follow - up interval for this subgroup of patients are needed. Furthermore, the affordability of health services is another critical factor in ensuring access and continuity of care for T2DM patients who need life - long follow - up, especially for those in lower - income populations [30, 31]. Financial support for additional out - of - pocket expenses, such as insurance coverage [32] or targeted subsidies [33], is necessary to facilitate the intensification of physician encounter frequency for patients with suboptimal control without burdening socioeconomically disadvantaged populations.

Existing evidence also suggested that encounter frequency could be reduced without compromising the intermediate health outcomes in cardiometabolic control among patients who have already achieved their treatment targets. Given the increasing burden on the health care systems, extending the follow - up interval of chronic patients has been suggested as one of the important means to improve the public’s access to health care [8]. An intervention program in the U.S. primary care settings investigated the feasibility of this idea, in which the proportion of the patients scheduled for ≥ 6 months had doubled in 2 years after the intervention, and the proportion of chronic patients achieving treatment targets also increased during the same time [34]. According to the Centers for Disease Control and Prevention in the U.S., patients with diabetes are encouraged to visit the doctor every 3 months if they have trouble meeting the treatment goals, and this interval is suggested to be extended to 6 months when the patients have achieved their treatment targets [35]. More scientific evidence is needed on the optimal extension in the encounter intervals for the patients with stable disease control, which is still controversial among the studies in this review. The inconsistency in the investigated follow - up intervals across the studies might be due to the differences in the medical settings (eg. primary care, specialized follow - up care in diabetes clinics / centers or outpatient follow - up in the hospitals), as well as the restriction in health resource and policy in different countries, which implied that context - specific studies are needed to find the optimized follow - up interval in individual healthcare systems.

Another major concern for extending the interval of the doctor consultation for patients with T2DM is whether the less - frequent encounters would impair the quality of diabetes care. The results of Asao’s study showed that less frequent encounters with doctors would not substantially impair the quality of diabetes care in terms of the necessary monitoring activities, at least for stable patients who do not need intensive monitoring [14]. Additionally, in the changing landscape of healthcare systems, the task delegation from physicians to non - physicians has been gaining popularity in diabetes care in recent years [36], and so has the development of technology for self - monitoring and point - of - care monitoring (e.g., HbA1c testing) [37, 38], which could also enable the extension of the physician encounter interval without compromising the quality of follow - up care in the near future. Additionally, considerable cost - saving for the less - frequent follow - up was reported in the studies on patients with stable disease control, especially for the indirect medical costs [13]. However, evidence on the cost - effectiveness of intensifying encounter frequency among patients during suboptimal control is still lacking. Given the small magnitude of the reported improvement in clinical performance, knowledge of the incremental cost - effectiveness of physician - encounter frequency is necessary to inform the decision - making in clinical practice and policymaking.

### Limitation in existing evidence and knowledge gaps for further studies

First, most studies were limited to small sample sizes, and some of the studies were restricted to the samples from a single center, which hampered the generalizability of the findings in these studies. Consequently, our review presents a valuable contribution by synthesizing evidence from various health systems, enabling a comprehensive understanding on the topic with enhanced generalizability. Second, only intermediate outcomes over a short follow - up period were investigated in most studies; evaluation of the long - term health outcomes, such as the incidence of cardiovascular diseases or other complications, is lacking in the current literature. So as the evidence on the cost - effectiveness, especially for patients with suboptimal disease control who may need to intensify the frequency of physician encounters. Third, systematic risk stratification is lacking in the existing studies of the optimal follow - up frequency, and the variance in the treatment targets in the studies makes it difficult to synthesize the existing evidence to generate recommendations for clinical practice. Additionally, to ensure comparability, only the studies on the follow - up visits involving physician encounters were included in our study. Given the transition to multi - discipline diabetes care models in the current climate, the optimal frequency of follow - up visits provided by other health professionals (if applicable in the specific health system) is also worth examining in the future. These issues need to be addressed in future studies to generate high - quality evidence to inform the optimization of physician encounters for such a large and highly heterogeneous population.

## Conclusion

Decisions on the frequency of physician - patient encounters among patients with T2DM have significant impacts on both patients’ health outcomes and the clinical burden on the health systems, whereas definitive intervals for physician - patient encounters are still lacking in most clinical guidelines. The findings of this systematic review suggested that the frequency of physician encounters could be individualized based on patients’ cardiometabolic control status. Specifically, physician encounter frequency could be reduced for patients with optimal cardiometabolic control without compromising the short - term health outcomes and quality of care; intensifying encounter frequency would benefit the patients with sub - optimal control, aiding in achieving treatment targets and improving quality of life. The findings of our reviews can help to inform not only clinical practice regarding the individualized care for diabetes patients, but also further research endeavors, particularly in the evaluation of the long - term health outcomes and the risk - based follow - up interval.

### Supplementary Information

**Additional file 1: Supplementary Figure 1.** Summary of risk of bias assessment for the included studies Randomized controlled trials (assessed by the Cochrane RoB2 tool).** Supplementary Figure 2.** Histogram plot for the findings in the sensitivity analysis after excluding the studies without specifying the type of diabetes^1^.

## Data Availability

No datasets were generated or analysed in this systematic review. All data extracted and used for the analysis was summarized in Table 1.

## Abbreviations

Pct.: Percentage
HbA1c: Hemoglobin A1C
SBP: Systolic blood pressure
DBP: Diastolic blood pressure
LDL: Low - density lipoprotein
HDL: High - density lipoprotein
TC: Total cholesterol
TG: Triglycerides
QOL: Quality of life
DMQLS: QoL Scale for Diabetes Mellitus
SF - 36: 36 - Item Short Form Survey (SF - 36) for quality - of - life measures
EQ - 5D: An instrument which evaluates the generic quality of life developed in Europe
JPY: Japanese Yen
diff: Difference
HR: Hazard ratio
T1DM: Type 1 diabetes
GDM: Gestational diabetes mellitus
