# Supplementary material for: Optimizing the frequency of physician encounters in follow - up care for patients with type 2 diabetes mellitus: a systematic review
Source: BMC Prim Care. 2024 Jan 26;25:41. doi: 10.1186/s12875-024-02277-9 (PMC10811944; doi:10.1186/s12875-024-02277-9)
Supplement: Supplementary file 1 — Additional file 1: Supplementary Figure 1. Summary of risk of bias assessment for the included studies Randomized controlled trials (assessed by the Cochrane RoB2 tool). Supplementary Figure 2. Histogram plot for the findings in the sensitivity analysis after excluding the studies without specifying the type of diabetes1. [file 12875_2024_2277_MOESM1_ESM.pdf]

## Supplementary method. Full search strategy in database

### MEDLINE Ovid search strategy

1 exp Diabetes Mellitus, Type 2/

2 ((type 2 or type II or type2 or typeII) adj2 diabet*).ti,ab.

3 (diabetes adj mellitus).ti,kw.

4 1 or 2 or 3

5 (frequen* adj4 (follow-up or visit* or revisit* or encounter* or consultation* or recall* or appointment* or attend* or schedul* or inspect* or check-up or examination* or monitor*)).ti,ab.

6 (interval* adj4 (follow-up or visit* or revisit* or encounter* or consultation* or recall* or appointment* or attend* or schedul* or inspect* or check-up or examination* or monitor*)).ti,ab.

7 (intensi* adj4 (follow-up or visit* or revisit* or encounter* or consultation* or recall* or appointment* or attend* or schedul* or inspect* or check-up or examination* or monitor*)).ti,ab.

8 (($month* or $week*) adj2 (visit* or revisit* or encounter* or consultation* or recall* or appointment* or attend* or schedul* or inspect* or check-up or examination* or monitor*)).ti,ab.

9 (($monthly or $weekly) adj2 follow-up).ti,ab.

10 5 or 6 or 7 or 8 or 9

11 4 and 10

12 exp animal/ not human/

13 11 not 12

**Embase Ovid search strategy**

1 exp Diabetes Mellitus, Type 2/

2 ((type 2 or type II or type2 or typeII) adj2 diabet*).ti,ab.

3 (diabetes adj mellitus).ti,kw.

4 1 or 2 or 3

5 (frequen* adj4 (follow-up or visit* or revisit* or encounter* or consultation* or recall* or appointment* or attend* or schedul* or inspect* or check-up or examination* or monitor*)).ti,ab.

6 (interval* adj4 (follow-up or visit* or revisit* or encounter* or consultation* or recall* or appointment* or attend* or schedul* or inspect* or check-up or examination* or monitor*)).ti,ab.

7 (intensi* adj4 (follow-up or visit* or revisit* or encounter* or consultation* or recall* or appointment* or attend* or schedul* or inspect* or check-up or examination* or monitor*)).ti,ab.

8 (($month* or $week*) adj2 (visit* or revisit* or encounter* or consultation* or recall* or appointment* or attend* or schedul* or inspect* or check-up or examination* or monitor*)).ti,ab.

9 (($monthly or $weekly) adj2 follow-up).ti,ab.

10 5 or 6 or 7 or 8 or 9

11 4 and 10

12 exp animal/ not human/

13 11 not 12

**Cochrane Library search strategy**

#1 MeSH descriptor: [Diabetes Mellitus, Type 2] explode all trees

#2 ((type 2 or type II or type2 or typeII) NEAR/1 diabet*) ti,ab

#3 (diabetes NEXT mellitus):ti,kw

#4 #1 or #2 or #3

#5 (frequen* NEAR/4 (follow-up or visit* or revisit* or encounter* or consultation* or recall* or appointment* or attend* or schedul* or inspect* or check-up or examination* or monitor*)):ti,ab

#6 (interval* NEAR/4 (follow-up or visit* or revisit* or encounter* or consultation* or recall* or appointment* or attend* or schedul* or inspect* or check-up or examination* or monitor*)):ti,ab

#7 (intensi* NEAR/4 (follow-up or visit* or revisit* or encounter* or consultation* or recall* or appointment* or attend* or schedul* or inspect* or check-up or examination* or monitor*)):ti,ab

#8 ((*month* or *week*) NEAR/2 (visit* or revisit* or encounter* or consultation* or recall* or appointment* or attend* or schedul* or inspect* or check-up or examination* or monitor*)):ti,ab

#9 ((*monthly or *weekly) NEAR/2 follow-up):ti,ab

#10 #5 or #6 or #7 or #8 or #9

#11 #4 and #10

## Supplementary Figure 1. Summary of risk of bias assessment for the included studies

### Randomized controlled trials (assessed by the Cochrane RoB2 tool)

| Study ID | D1 | D2 | D3 | D4 | D5 | Overall |
|---|---|---|---|---|---|---|
| Wermeling, 2014 | ! | + | + | + | + | ! |
| Wermeling, 2013 | ! | + | + | + | + | ! |
| Hu, 2012 | + | + | + | + | + | + |

+ Low risk

! Some concerns

- High risk

| | |
|---|---|
| D1 | Randomisation process |
| D2 | Deviations from the intended interventions |
| D3 | Missing outcome data |
| D4 | Measurement of the outcome |
| D5 | Selection of the reported result |

### Cohort studies (assessed by the Newcastle-Ottawa Scale)

| NOS criteria | Studies | | | | | | | | |
|---|---|---|---|---|---|---|---|---|---|
| | Zhao, 2022 | Ye, 2021 | Ukai, 2019 | Dobbins, 2019 | Moradi, 2017 | Asao, 2014 | Egan, 2012 | Morrison, 2011 | Turchin, 2010 |
| A. Selection (maximum of four stars) | | | | | | | | | |
| 1. Representativeness of the exposed cohort | | ★ | ★ | ★ | | ★ | ★ | | |
| 2. Selection of the non-exposed cohort | ★ | ★ | ★ | ★ | ★ | ★ | ★ | ★ | ★ |
| 3. Ascertainment of exposure | ★ | ★ | ★ | ★ | ★ | ★ | ★ | ★ | ★ |
| 4. Demonstration that outcome of interest was not present at start of study | ☆ | ★ | ★ | ★ | | | | ★ | ★ |
| B. Comparability (maximum of two stars) | | | | | | | | | |
| 1. Comparability of cohort on the basis of the design or analysis** | | | | | | | | | |
| a). Study control for age | ★ | ★ | ★ | ★ | | ★ | ★ | ★ | ★ |
| b). Study control for additional factors | ★ | ★ | ★ | ★ | | ★ | ★ | ★ | ★ |
| C. Outcome (maximum of three stars) | | | | | | | | | |
| 1. Assessment of outcome | ★ | ★ | ★ | ★ | ★ | ★ | ★ | ★ | ★ |
| 2. Was follow-up long enough for outcomes to occur** | ★ | ★ | | ★ | ★ | | ★ | ★ | ★ |
| 3. Adequacy of follow-up of cohorts | ★ | ★ | ★ | ★ | ★ | ★ | ★ | ★ | ★ |
| Total (maximum of nine stars) | 7 | 9 | 8 | 9 | 5 | 7 | 8 | 8 | 8 |

Notes:

* For the analyses on the outcomes of interest listed in Table 1

** 2-year follow-up would be considered as long enough for the observation of blood glucose and cardiometabolic control.

☆: Not applicable

**Supplementary Figure 2. Histogram plot for the findings in the sensitivity analysis after excluding the studies without specifying the type of diabetes[1]**

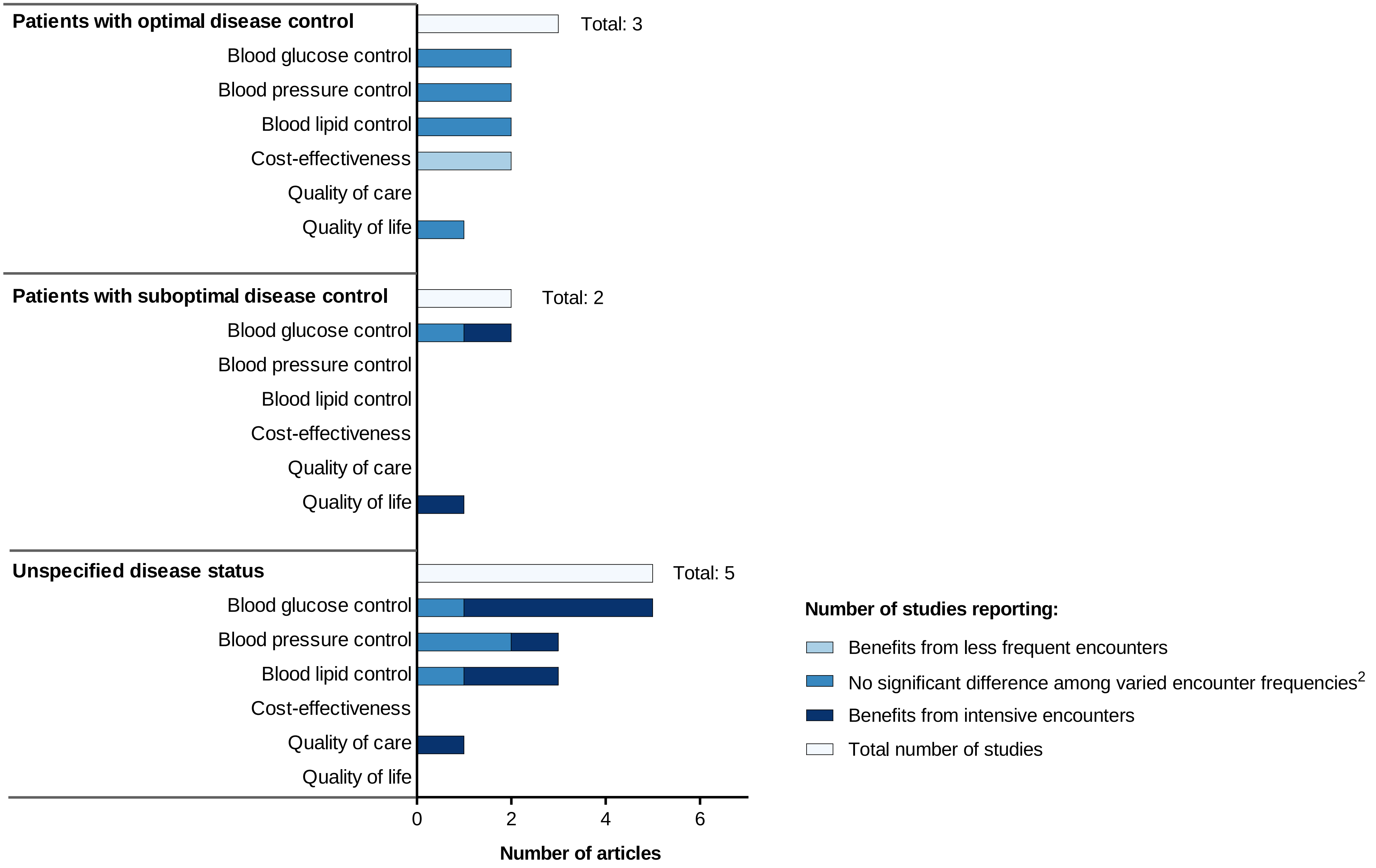


Notes:
1. Studies excluded in this sensitivity analysis: Morrison, 2011; Turchin, 2010.
2. Studies reported no significant difference in the outcome of interest among the patients with varied encounter frequencies.
